# Video Q & A: Alcohol - a global problem. An interview with Ian Gilmore

**DOI:** 10.1186/s12916-014-0209-z

**Published:** 2014-10-21

**Authors:** Ian Gilmore

**Affiliations:** University of Liverpool, Liverpool, L69 3BX United Kingdom

**Keywords:** Alcohol, Global health, Addiction, Psychiatry

## Abstract

In this video Q & A, we talk to Ian Gilmore about the global burden of alcohol in terms of its scale, and its consequences in relation to non-communicable diseases and other illnesses. Sir Gilmore additionally discusses how the problem is being addressed, reviews the outcomes of recent initiatives and describes the challenge and future directions.

**Electronic supplementary material:**

The online version of this article (doi:10.1186/s12916-014-0209-z) contains supplementary material, which is available to authorized users.

## Introduction

Professor Sir Ian Gilmore is an honorary consultant physician at the Royal Liverpool University Hospital and holds an honorary chair at the University of Liverpool. After training in Cambridge, London and the USA, he moved to Liverpool as a consultant in 1980, where he has enjoyed working ever since. His specialty interest is liver disease. He is the immediate past-president of the Royal College of Physicians (RCP) and is currently president of the British Society of Gastroenterology. He is chairman of Liverpool Health Partners, an organisation created between the University and the teaching hospitals in Liverpool to promote an Academic Health Science Centre in order to foster academic innovation, education and service development in the region. He has particular interest in health harms related to alcohol misuse and the role of regulation in reducing this. He chaired a RCP Working Party in 2001, producing the report “Alcohol - can the NHS afford it? A blueprint for a coherent alcohol strategy”. He chairs the UK Alcohol Health Alliance, in which relevant agencies work together in a coherent and focused framework. He has also been appointed as Chair of the European Alcohol and Health Forum Science Group.

In this interview (Additional file 1), we talk to Prof Sir Ian Gilmore about the global alcohol problem and the harms it causes. We discuss how policies are tackling the issue and what are the difficulties associated with embracing the strategies.

## Edited transcript

### (1) What is the scale of the alcohol problem?

The global scale of the alcohol problem is really quite remarkable. One cannot help feeling that if it was another infectious disease, like that caused by the Ebola virus, it would get a much higher priority. However, we are also comfortable with alcohol as it is much entrenched in our day-to-day lives such that it is very hard to persuade people of the magnitude of the problem. Of all the continents, Europe is the heaviest consuming continent. If you look at alcohol consumption in Russia over the last ten or 15 years, it has decimated the male population in the way that HIV did in sub-Saharan Africa. So it really is a huge problem. I think the life expectancy of males in Russia fell by about ten years when Boris Yeltzin became the president of the Russian Federation and they de-regulated the availability of alcohol.

The increase in consumption we have seen in the UK over the last 20 to 30 years is not unique. For instance, the consumption curve of alcohol over the last 20 years in China, mirrors what is seen in the UK, but it starts at a much lower level. However, there’s no doubt that the global industry is moving into developing countries, and some of the social norms there will change. I think we will see the consumption of alcohol in women in these countries catching up with their European counterparts over the next 10 to 30 years. There is a narrowing of the differences in alcohol consumption between countries due to more travel and as the multinational companies push their same policies throughout the world.

### (2) Can you describe the consequences in relation to non-communicable diseases and related illnesses?

Alcohol is a risk factor in many non-communicable diseases including cirrhosis, hypertension, stroke and various cancers. The WHO has shown that the biggest single global risk factor of a man dying before the age of 60, or having disability-adjusted life years is from alcohol first as compared to that from risk factors such as tobacco, unsafe sex, diabetes and hypertension. This is really because alcohol tends to kill people relatively young. It tends to be when people are in the most productive phase of their life. Tobacco clearly kills more people overall, but much more often in their later life. Thus alcohol is a key risk factor in productivity in the workforce.

The scale of the problem we are seeing from alcohol is huge globally, and also in individual countries like the UK. There are a range of harms. At one end of the spectrum there are the harms from getting drunk, that are seen in young people who are binge drinking. These dangers include violence, accidents, unwanted pregnancies and basically losing control. At the other end of the spectrum, there are the consequences of long-term chronic consumption. This tends to be seen more in older populations and causes cirrhosis of the liver, cancers and hypertension.

Besides the young person/old person divide observed in the spectrum there is a gender divide. If you go back several decades, it was probably socially unacceptable to see a woman drinking in a pub or bar. However now it seems to be socially acceptable for women to be lying in the street on a saturday night and highlights a cultural change.

It is interesting that young people drink less often than older people, however when they drink, they consume more alcohol. Older people drink less alcohol on any one occasion, but are likely to drink more frequently. Many 50 to 70 year-olds are drinking on a nightly basis. Probably about a quarter of the population over 65 will drink every day or six days a week. Many of them will come to no harm, but there are increasing numbers of older people coming into hospitals with head injuries, falling down the stairs when they have been less than steady late in the evening. In young people, there are tragedies such as paraplegias and quadriplegias when they dive into shallow swimming pools in the small hours of the morning and break their necks. Thus overall there is a spectrum of harm due to alcohol consumption that varies between the young and the old and between men and women.

### (3) How is the problem being addressed in terms of policies and strategies?

There is a very strong evidence base demonstrating what strategies that work. We know that the amount of harm you see in a population, whether it is in a village, city, country or continental level is related to how much alcohol is consumed and the evidence shows that that the more a population drinks, the more harm it sees. We know that the drivers of how much harm we see are basically price, availability and marketing. Of those three, price is probably the single most important factor. It is like cigarettes and if you plot consumption against price, there is a very tight inverse relationship.

There are policy tools but these tools are not always very popular with governments. This is in part because they see alcohol as one of the pleasures of the voter, and they are worried about losing votes if they implement policies such as putting up the price or banning advertising. Additionally, recent publications clearly indicate that they are strongly influenced by the drinks industry. The drinks industries have become global and multinational, with an absolutely huge influence.

Now in the UK, for example, it is not just the producers but also the retailers and the supermarkets that are perhaps the main cause of the large increase in drinking at home. This is due to drinking cheap supermarket alcohol beverages. People in the UK now do not want to spend seven or eight pounds on a single drink when they can buy the whole bottle of alcohol and drink it at home. This has led to a change of culture. The drinks industry, when they have debated with me, say, “This is nothing to do with price or availability –this is an issue of culture”. Well, culture in the UK has changed completely in the last 10–20 years because of the availability of cheap off-licence supermarket drink. If you think back to 10–20 years ago, most alcohol was consumed in pubs, bars and restaurants. Now for everything except beer, about 75% or more of alcoholic beverages are consumed at home.

This does not mean to say that the person necessarily stays at home and drinks alcohol. We have a culture of front-loading or pre-loading, where young people will, so to speak, “tank up” on alcohol before they go out, and then they will just keep topped up with a small amount of alcohol at the more expensive prices. Or even now people talk about “side-loading”, which is where people go to an off-licence to buy a cheap bottle of alcohol to top up, and then go on with their night out between pubs, bars and clubs.

### (4) Are there any outcomes from recent initiatives?

I think there is a huge variability across the world as to how much they have embraced the evidence on price, on availability and on marketing. There are some shining examples, for example France, that historically have had very high levels of consumption and harm, have seen dramatic falls in the last 20 to 30 years. This is not solely because of regulation but there have been changes in culture there. This includes the measures that have been adopted like having a complete ban on alcohol companies sponsoring sports events and music festivals. They have also had a complete ban on broadcast advertising and these have almost certainly been important. In contrast, if you come to the UK, there are alcohol adverts at half time in premier soccer games showing visual cues on alcohol drinking. Youngsters see something to do with alcohol every 20 seconds, either on the players’ shirts or on an advertisement on a billboard round the grounds. Thus France has really been quite tough and the UK has a remarkably lax regulatory framework.

If you go to developing countries, obviously it is even less well regulated. There are worrying developments where some alcohol producers are now going around the world offering to help countries write their alcohol strategies, which I have to say does not strike me as being the most sensible public health policy. In the UK, we had a glimmer of hope that there would be a real evidence-based policy instituted when the Prime Minister, David Cameron, came out in favour of a minimum unit price. This is a minimum floor price, where you cannot sell alcohol per unit strength below a certain amount. It is a very attractive policy because not only does it tackle cheap drink, but it targets the people you want to target most because a minimum floor price tackles the cheapest alcohol. We know that the heaviest drinkers and the underage drinkers gravitate to that cheapest drink.

A lot of the evidence around it was circumstantial and modelling of minimum unit price. However, in the last two to three years, some really important data is coming out of Canada, where in some states like Saskatchewan they have in effect had a minimum floor price for some years. When they increased that floor price by 10%, they actually saw a dramatic fall in alcohol-related harm. Indeed they saw a 30% fall in deaths directly attributable to alcohol. So we now have got real life evidence that a minimum unit price is a highly effective policy. Unfortunately, the present government has let it roll into the long grass for the moment, due to the concerns of voters and from the pressure from the drinks industry.

### (5) What do you think are the current challenges?

I think the challenge facing public health is to get governments to embrace these evidence based policies. Perhaps in order to help governments, I think we also have to try and get the general public onside. I think if you stop someone in the street on a friday evening and ask them if they would like to pay more for their weekend bottle of wine, they are not going to say yes. However, there is evidence that if it is put in the context of the harm that their local communities are seeing from alcohol then you get a different answer. There is hardly a family that has not been affected by alcohol in some way, whether it is children of dependent parents, or a victim of violence, or a whole manner of harms that we see. It is interesting that it was actually the debate about passive smoking (non-smokers being exposed to other peoples’ smoke) that really swung the argument behind banning smoking in public places. Of course the harm to third parties is much greater from alcohol misuse. I think this is a message we need to pick up on and mobilise public opinion.

### (6) Can you discuss the future directions for this global problem?

I think it is very difficult to know where we are going with the alcohol problem. We are seeing different patterns in different countries. On the other hand, there is also perhaps a convergence as these multinational companies become dominant – so they are drinking the same in the bars of Mumbai as they are in New York and London. Of course, in developing countries there is still a huge problem in rural areas, where local spirits are distilled and local beers are produced that are often contaminated and cause harm through that route. I do not quite know how it is going to play out.

I think in all countries we have a rather ambivalent attitude towards alcohol. It is quite hard to talk about alcohol without smiling, because we associate it with having a good time. It is difficult to envisage a celebration in the UK now without alcohol being in the centre. You do not get that same sort of attitude around smoking; most smokers, in their heart of hearts, know that they are addicted and would like to stop if they could. Most drinkers do not want to stop. I think it is that sort of cultural barrier that we have to get through.

Of course, we do not want everybody to stop drinking. That is not the endgame. However, nonetheless, if we could just get the whole consumption curve shifted down by 10 or 20% - if we could get back to where we were in the UK, say, 30 to 40 years ago in our per capita consumption and we would see a huge benefit in health.

I think one of the difficulties we have is mobilising public opinion. And I think public health doctors need to develop a stronger voice. One problem I encountered was that there were lots of organisations that were concerned about the impact of alcohol on health, but they were not necessarily singing from the same hymn sheet. Therefore, about five years ago, we formed the Alcohol Heath Alliance in the UK, which is now made up of more than 35 organisations which are all independent of the drinks industry and who all are concerned about the level of harm we are seeing from harmful alcohol use. It is a very loose alliance and we do not interfere with individual organisations’ right to say what they like. But I think in fact we by and large see the same problems and would like to see the same policy changes. By coming together, we are able to make sure that we are singing in unison – or at least like a choir, in harmony – rather than crossing each other.

The World Health Organisation has undoubtedly been a leader in the field of alcohol, in terms of developing model strategies of plotting the levels of harm in different countries. However, the WHO resources are very limited and I think we really need to get the United Nations more involved as they did with tobacco. We need to get the harm from alcohol to communities worldwide high on the UN agenda.

### (7) Where can I find out more?

See references [1-10].

## Additional file

Additional file 1:
**An interview with Ian Gilmore.**
